# Composition Restoration Enables Recycling of Mixed‐Cation, Mixed‐Halide Perovskites for Solar Cells

**DOI:** 10.1002/adma.73266

**Published:** 2026-05-05

**Authors:** Zhenni Wu, Katharina Dammer, Robert Skunde, Mykhailo Sytnyk, Christian Göllner, Fei Ding, Juan S. Rocha‐Ortiz, Albert These, Balázs Imre, Yanxue Wang, Dorothea Wisser, Christoph Brabec, Ian Marius Peters

**Affiliations:** ^1^ Department of Material Science Institute of Materials for Electronics and Energy Technology (i‐MEET) Friedrich‐Alexander‐Universität Erlangen‐Nürnberg Erlangen Germany; ^2^ Helmholtz Institute Erlangen‐Nürnberg for Renewable Energy (HI ERN) Forschungszentrum Jülich Erlangen Germany; ^3^ Erlangen Center for Interface Research and Catalysis (ECRC) Friedrich‐Alexander‐Universität Erlangen‐Nürnberg Erlangen Germany

**Keywords:** Recycling, perovskite, solar cells, lead, composition

## Abstract

The rapid industrial emergence of perovskite photovoltaics (PV) highlights their potential to complement silicon PV in meeting the growing global solar demand. As deployment scales, closed‐loop recycling of perovskite PV will be beneficial to conserve critical resources and mitigate environmental risks associated with lead. However, mixed‐cation, mixed‐halide perovskites—typical in record‐efficiency devices—undergo systematic composition drift during device fabrication. Consequently, material recovered from end‐of‐life modules inherits these deviations, degrading cell performance if reused without adjustment. To overcome this fundamental bottleneck in circular manufacturing, we developed a comprehensive quantification framework to audit and restore perovskite composition. By combining nuclear magnetic resonance (NMR), inductively coupled plasma‐optical emission spectroscopy (ICP‐OES), and ion chromatography (IC), we obtained full compositional fingerprints of the hybrid perovskite recovered from processed solar‐cell stacks, allowing us to resolve their altered composition and restore the material to match the original precursor formulation. Composition restoration effectively closed the performance gap, yielding recycled perovskite cells with efficiencies comparable to pristine devices. A cost analysis demonstrates this approach can achieve a 69.1% cost reduction, while preserving supply‐constrained elements like Cs and I. These results demonstrate a practical, compositionally informed pathway for the sustainable, closed‐loop manufacturing of complex perovskite absorbers.

## Graphical abstract

Compositional drift during processing prevents the direct recycling of mixed‐cation, mixed‐halide perovskites. This work introduces a framework to quantitatively audit and restore recovered materials, enabling closed‐loop recycling with efficiencies comparable to virgin perovskites. This approach can achieve a 69.1% cost reduction, establishing a sustainable and economically favorable blueprint for manufacturing complex perovskite absorbers.

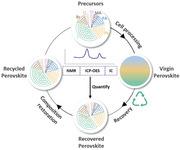

## 1 Introduction

Perovskite photovoltaics (PV) have emerged within just two decades as a leading next‐generation solar technology, and the rapid advent of companies in perovskite PV [1] highlights their potential to complement conventional silicon photovoltaics in achieving the global solar capacity expected to reach tens of terawatts by 2050 [2, 3, 4]. Perovskite/silicon tandem photovoltaics are projected to account for approximately 10% of the solar market by 2050 [5, 6], or up to 35% under more aggressive growth scenarios [5]. Recycling of perovskite photovoltaics will be beneficial for recovering valuable raw materials and for addressing the environmental impacts of resource extraction, as well as for supporting the growth of the industry. Equally important, recycling provides a pathway to safely manage lead [7], an essential yet environmentally concerning component of all high‐efficiency perovskite devices [8].

Researchers have developed various approaches to recycle lead from perovskite solar cells. In general, the perovskite is first dissolved in organic solvents, after which lead is extracted using agents such as aqueous ammonia [9], iron‐incorporated hydroxyapatite composites [10], or ion‐exchange resins [11]. The recovered lead is subsequently converted into PbI_2_ for reuse in perovskite solar cells (PSC) fabrication.

Yet, lead is not the only element that merits recycling. Recycling the entire perovskite compound is often preferable, as it realizes 100% atomic recycling, simplifies reprocessing for subsequent device fabrication and allows recovery of elements with greater economic value than lead, thereby strengthening the incentives for perovskite PV recycling [12]. Among these, cesium (Cs) and iodine (I) are particularly critical. The current market for Cs is small, and its production is limited, which may not meet the growing demand projected for perovskite PV in the future [13]. Iodine constitutes the largest fraction of perovskite composition, yet it is one of the scarcest non‐metallic elements [14]. Its primary sources are marine organisms and brines, while the planet's largest reserve—seawater— remains uneconomical to exploit due to extremely low concentrations [15]. Furthermore, about half of the global iodine production is consumed in health‐related applications, for which no substitutes exist [15]. Compounding the issue, 91% of iodine production is concentrated in Chile and Japan, leaving supply vulnerable to local policy decisions and environmental disruptions, which in turn drive significant price volatility [15].

Several studies have extended recycling efforts to the entire perovskite compound, including methylammonium (MA)‐ and formamidinium (FA)‐based lead halides such as MAPbI_3−x_Br_x_ [16], MAPbI_3_ [12], and FAPbI_3_ [17]. It should be noted that they all focus on mono‐cation perovskites, while the recycling of multi‐cation, mixed‐halide perovskites has not yet been investigated. However, record device efficiencies are predominantly achieved with these more complex compositions, primarily FAMACs‐based perovskites [18, 19, 20].

The main challenge in recycling mixed‐cation, mixed‐halide perovskites is the deviation in film composition from the precursors after cell processing, particularly when Cs is involved—likely caused by the lower solubility and limited volatility of Cs species than the organic cations [21]. Recycling targets the perovskite film in a solar cell, whose composition already deviates from that of the precursors. As a result, the composition of the recovered perovskite is expected to differ from the initial input. However, the composition of the perovskite precursor solution is strongly correlated with device performance [22], and even minor variations (0.5%–1%) can lead to significant changes in perovskite film properties and overall cell performance [23]. This suggests that even perfect recovery of a mixed‐cation, mixed‐halide perovskite film—i.e., complete preservation of the film composition—would not enable direct reuse of the recovered material in subsequent device fabrication. Thus, composition drift of hybrid perovskites during processing is a structural barrier for closed‐loop recycling of technologically relevant absorber materials.

Rectifying the composition of the recovered perovskite would be necessary to match the initial composition and thereby restore device performance. This, however, requires accurate knowledge of the full composition of the recovered material. Yet, reported methods for determining perovskite film composition are scarce and largely limited to cation quantification by solid‐ or solution‐state nuclear magnetic resonance (NMR) [21, 24, 25]. One study determined all components of FA_m_MA_1−m_Pb(I_x_Br_y_Cl_z_)_3_ perovskite films except chlorine by deconstructing the film into organic (FAI, MAI, and MABr) and inorganic (PbI_2_) components with ethanol and water, respectively, followed by analysis with UV–vis spectroscopy [26]. Nevertheless, a methodology that additionally quantifies Cs and Cl—both key elements in many high‐efficiency cells—has not yet been demonstrated. To establish a generalizable quantification framework for hybrid perovskites, particularly within recycling contexts, it is essential that the quantification be comprehensive, preferably encompassing all cations (FA, MA, and Cs), halides (I, Br, and Cl), and potential impurities originating from other layers.

In this work, we report a recycling strategy for a mixed‐cation (FA, MA, and Cs), mixed‐halide (I, Br, and Cl) perovskite that integrates composition restoration. We first established an approach to recover the hybrid perovskite directly from precursor mixtures, thereby avoiding composition deviations introduced during cell processing. We then developed a comprehensive quantification method to determine the elemental composition of hybrid perovskites. Finally, the method was applied to the hybrid perovskite recovered from a device stack, whose composition was then restored to match that of the original precursors. The composition‐corrected recycled perovskite was subsequently used to fabricate solar cells, which delivered efficiencies comparable to those based on the virgin material.

## 2 Results and Discussion

**FIGURE 1:**
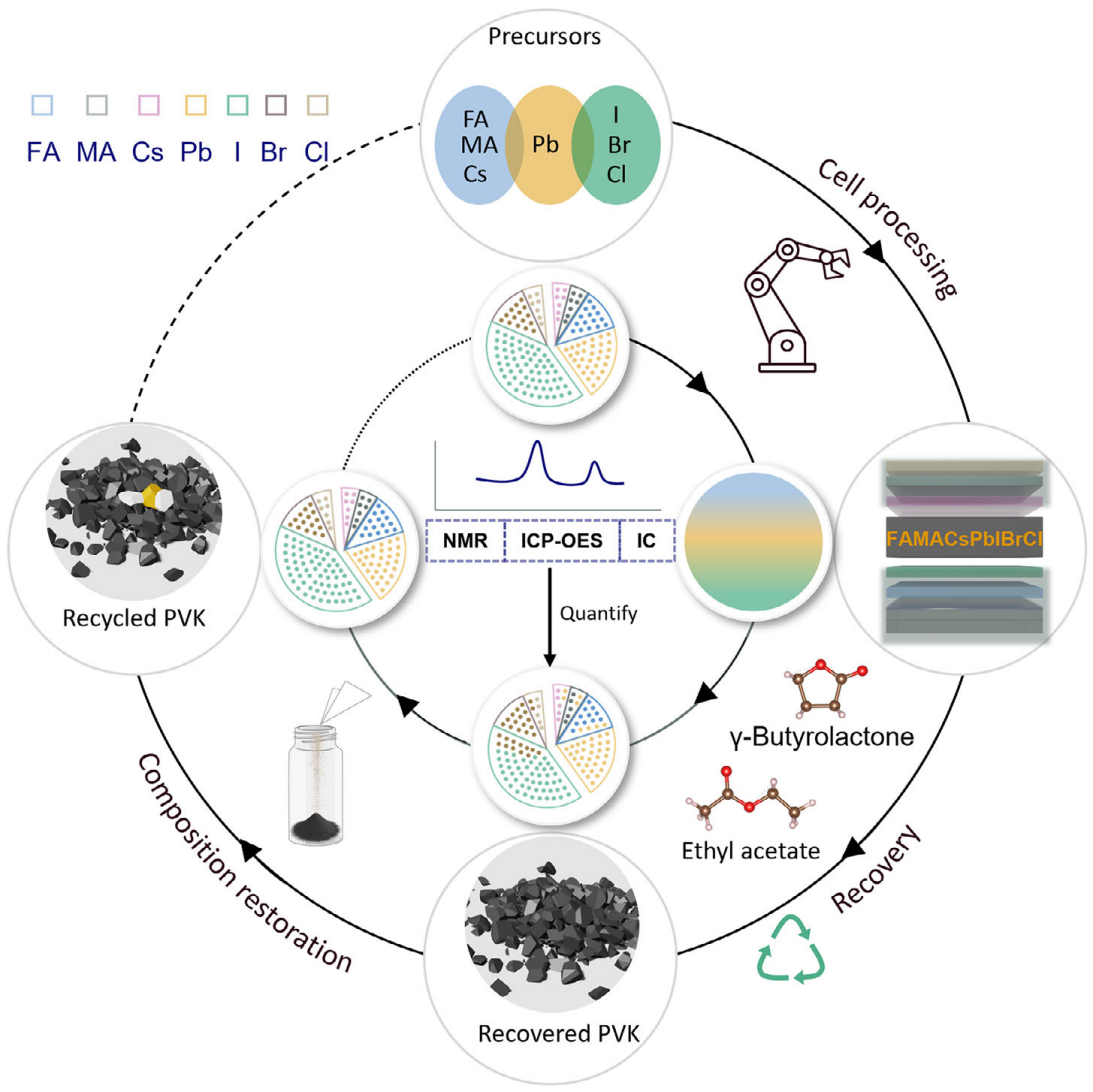
Schematic illustration of the life cycle of the investigated mixed‐cation, mixed halide perovskites (PVK)—from the precursors to the recycled perovskites suitable for reuse in solar cells—showing the composition evolution throughout the process.

Figure 1 illustrates the life cycle of the investigated mixed‐cation, mixed‐halide perovskite, highlighting the compositional evolution throughout the process. The workflow begins with evaluating how cell processing affects the perovskite film composition, followed by recovering the hybrid perovskite from solar devices. A quantitative analysis was then established to determine the composition of the recovered material, enabling its restoration to the precursor composition. The restored perovskite was subsequently reused for solar cell fabrication, demonstrating the circular use of the complex absorber.

### 2.1 Complexity of Recycling Multi‐Cation Perovskites: Compositional Change During Cell Processing

**FIGURE 2:**
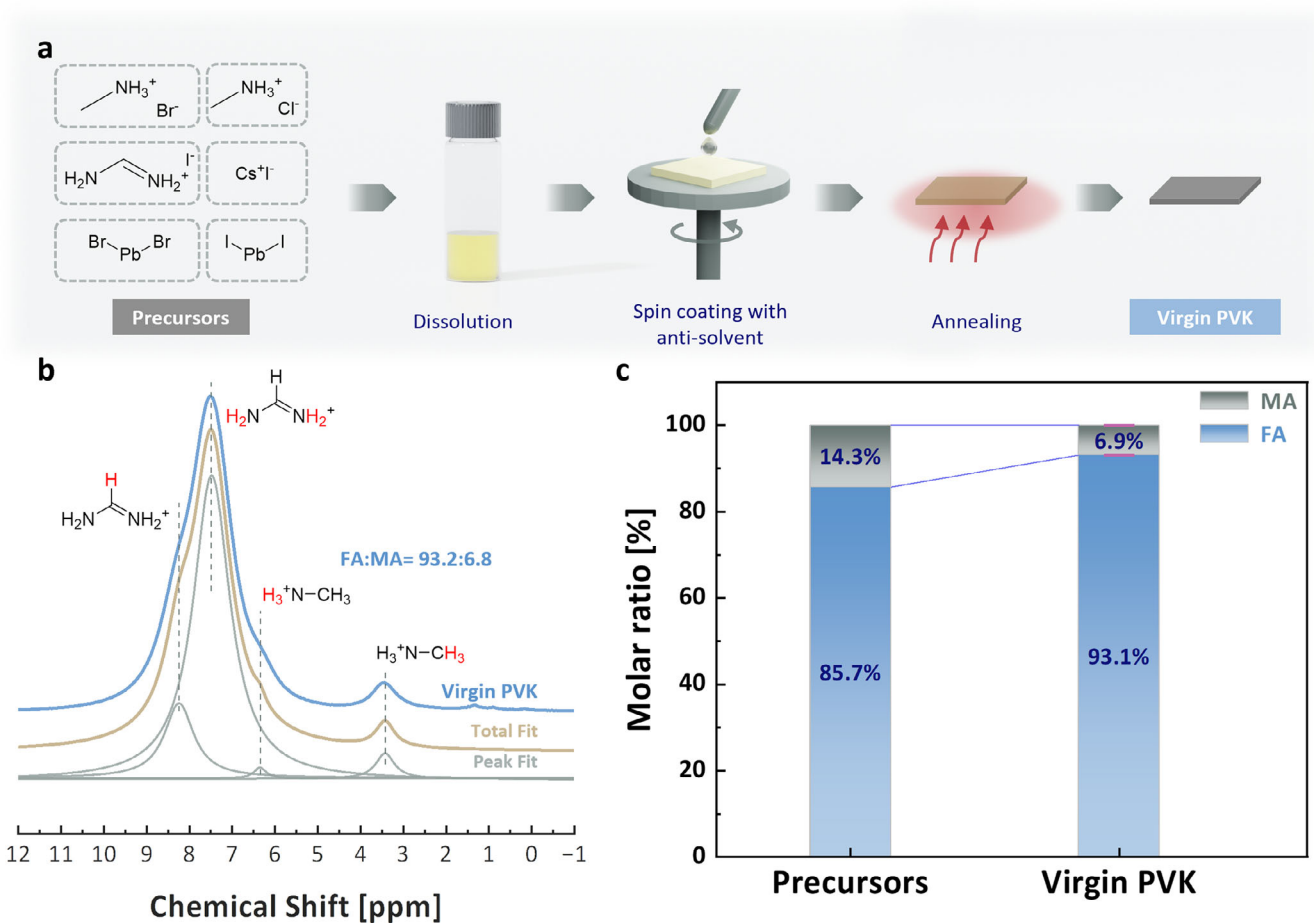
Evolution of FA:MA ratio from precursor salts (“Precursors”) to the as‐deposited perovskite (“Virgin PVK”). (a) Schematic of the conversion from precursors to Virgin PVK. (b) ^1^H MAS NMR spectrum of Virgin PVK with peak deconvolution. (c) FA:MA molar ratio of Virgin PVK derived from their ^1^H MAS NMR, compared with that of the precursors; the error bars (marked in red) for Virgin PVK show variability across 3 independent samples (*n* = 3).

The mixed‐cation, mixed‐halide perovskite, selected from literature [27], was prepared from commonly used precursor salts: CH_3_NH_3_Br (MABr), CH_3_NH_3_Cl (MACl), CH(NH_2_)_2_I (FAI), CsI, PbBr_2_, and PbI_2_. The nominal composition, based on the precursors, is FA_0.877_MA_0.146_Cs_0.054_PbI_2.838_Br_0.138_Cl_0.1_. To determine whether this composition changes during device fabrication, we prepared perovskite films using the same procedures as for cell fabrication (Figure 2a). The molar ratio of FA to MA in the resulting films was analyzed by ^1^H Magic Angle Spinning NMR (MAS NMR) (Figure 2b). A complementary ^13^C MAS NMR spectrum, confirming the presence and chemical nature of both cations, is provided in Figure S1. Deconvolution of the proton spectrum yielded an FA:MA ratio of 93.2:6.8, deviating notably from the nominal ratio of 85.7:14.3. Duplicate samples showed highly consistent results (Figure 2c). This implies the loss of MA during cell processing. Annealing has been identified as a step where such loss can occur [28].

### 2.2 Recovery Strategy for the Mixed‐Cation, Mixed‐Halide Perovskite

As cell processing alters the perovskite composition, we first evaluated the feasibility of recovering the perovskite directly from the precursor salts, excluding all cell‐processing steps and non‐perovskite layers to eliminate compositional variations. After establishing this proof of concept, we then extended the approach to recover the hybrid perovskite from solar‐cell stacks.

**FIGURE 3:**
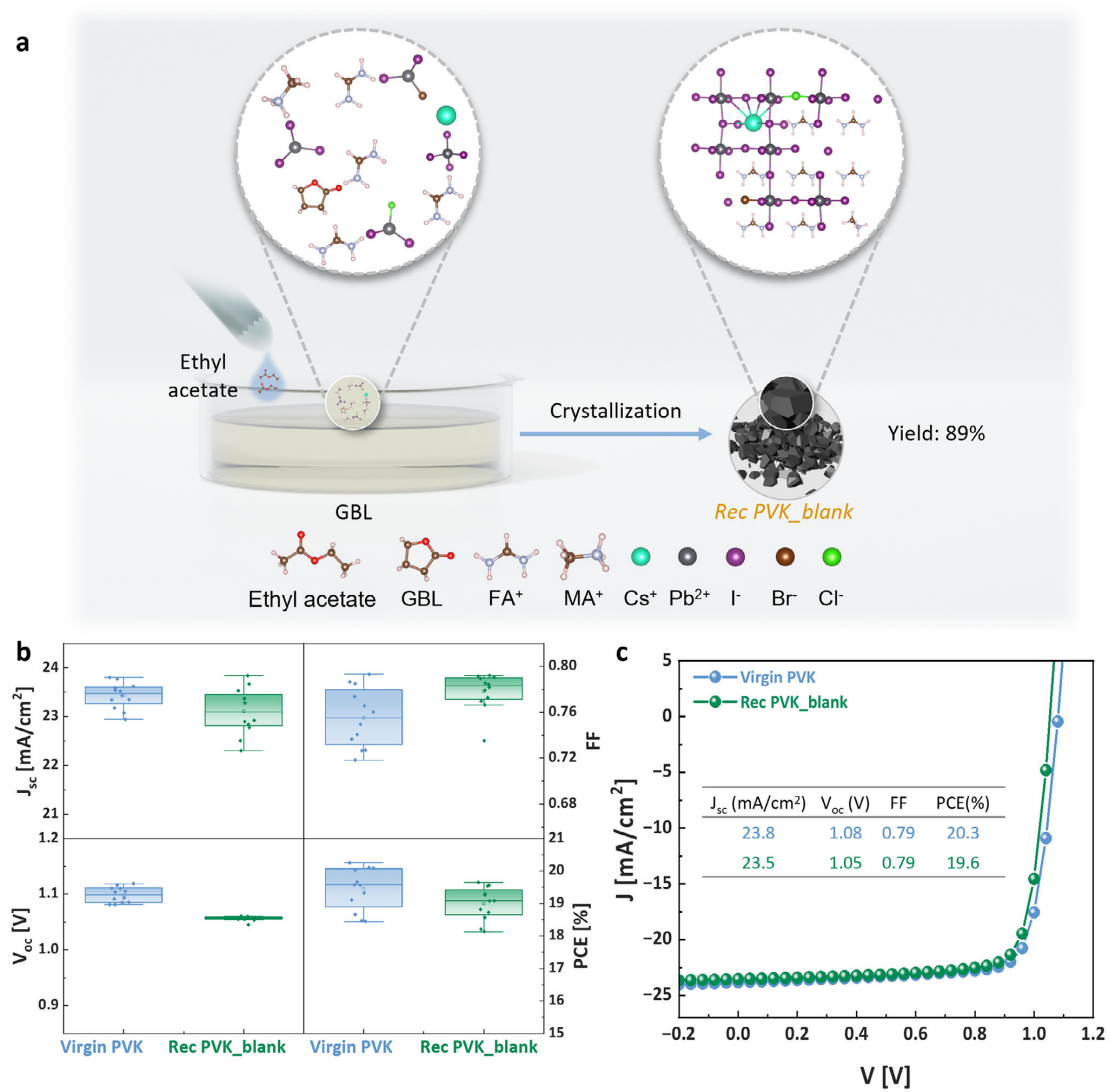
Direct recovery of the mixed‐cation, mixed‐halide perovskite from precursor salts, excluding cell‐processing steps. (a) Schematic of the recovery process for the hybrid perovskite via anti‐solvent crystallization. (b) Photovoltaic metrics (J_sc_, V_oc_, FF, and PCE) of solar cells fabricated from pristine chemicals only (“Virgin PVK”) and recovered perovskite (“Rec PVK_blank”), the latter recovered directly from the precursor salts. (c) *J*–*V* characteristics of the best‐performing devices based on Virgin PVK and Rec PVK_blank.

We used anti‐solvent crystallization as the recovery approach (Figure 3a). Among common perovskite solvents (N,N‐dimethylformamide (DMF), Dimethyl sulfoxide (DMSO), 2‐methoxyethanol, N‐Methyl‐2‐pyrrolidone (NMP), and GBL), we selected GBL for its greener profile and a low Gutmann's donor number. A lower donor number implies weaker coordinating ability to Pb^2+^ and thus fewer solvent–Pb adducts than for stronger donors such as DMSO, NMP, or DMF [29]. Due to the weak Lewis basicity of GBL, halides outcompete the solvent for Pb^2+^, forming halo‐plumbate species, such as PbX_3_^−^, PbX_4_^2^^−^ (X = I, Br, and Cl), facilitating subsequent crystallization [29]. The A‐site cations (FA^+^, MA^+^, and Cs^+^) primarily associate with these anions as contact ion pairs. Additionally, N─H groups of FA^+^ and MA^+^ can form hydrogen bonds to the GBL carbonyl. Ethyl acetate served as the anti‐solvent. Upon ethyl acetate addition, the dielectric constant would decrease sharply, reducing ion stabilization and driving supersaturation and crystallization. Ethyl acetate is also a weaker hydrogen bond acceptor due to steric hindrance and delocalized electron density [30]. Consequently, ethyl acetate dilutes GBL and competes for solvation, weakening the hydrogen bond between N─H and the GBL carbonyl, further promoting crystallization. After crystallization, the product was washed twice with ethyl acetate to remove residual GBL. This recovery approach resulted in an overall perovskite yield of 89%. The observed material loss is primarily attributed to mechanical stages such as centrifugation and washing. While a fraction of perovskite may remain in the mother liquor (ethyl acetate/GBL), this Pb^2+^‐containing liquid stream can be managed within a sustainable, closed‐loop framework. For instance, hydroxyapatite—a biocompatible material mimicking human bone—has been demonstrated to sequester lead ions from organic waste, reducing concentrations to ppb levels [10, 31]. Integrating such established lead‐mitigation protocols would further support the environmental responsibility of the recycling process.

The perovskite recovered from the precursor salts with this approach is denoted as “Rec PVK_blank”. Solar cells prepared with Rec PVK_blank showed performance comparable to that of the virgin devices (Figure 3b). A Welch's *t*‐test—which accounts for unequal variances—was performed on their power conversion efficiencies (PCEs) (Table S1), revealing no statistically significant difference between the two groups (*p* > 0.05). This result indicates that the recovery strategy is effective when compositional changes during cell processing are either avoided—as in Rec PVK_blank– or subsequently corrected.

The best‐performing device with Rec PVK_blank reached 19.6%, compared with 20.3% from the champion device using virgin perovskite (Figure 3c). The small performance deficit arises from a reduced V_oc_, as also visible in the box plot. Introducing a thin phenethylammonium iodide (PEAI) passivation layer to the device structure—as a diagnostic interlayer—eliminated this V_oc_ difference for both materials (Figure S2), indicating that the effect is interface‐addressable rather than intrinsic to Rec PVK_blank.

**FIGURE 4:**
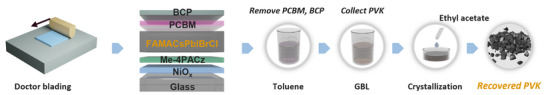
Workflow for recovering the mixed‐cation, mixed‐halide perovskite from solution‐processed device stacks.

We next applied the method to the recovery of perovskite from solution‐processed device stacks. The overall workflow is summarized in Figure 4. For this work, we prepared functional perovskite solar cells by spin coating; however, spin coating typically wastes ∼90% of the dispensed ink [32]. To obtain an adequate amount of perovskite for recovery, we established a parallel route—adapted from our previous work [10]– that generated blade‐coated pseudo‐modules with the same structure as the spin‐coated cells, excluding indium tin oxide (ITO) and Ag layers. To match film chemistry, we used the same solvent mixture for the perovskite solution (DMF:DMSO, 4:1 v/v). We then recovered the perovskite from the pseudo‐modules by removing [6,6]‐Phenyl‐C61‐butyric acid methyl ester (PCBM) and Bathocuproine (BCP) via toluene immersion, dissolving the perovskite film off the stack with GBL, and inducing crystallization with ethyl acetate to obtain recovered perovskite. Toluene immersion is highly selective and does not alter the perovskite composition, as toluene is a nonpolar antisolvent for perovskites. It lacks the polarity required to stabilize separated ions or break the strong Coulombic attractions and hydrogen bonding within the perovskite lattice or potentially residual organic halide precursors. Such resilience is consistent with existing literature, which confirms that the perovskite's morphology and crystal phase remain unaltered during extended one‐week immersion in toluene [33]. While minor physical detachment of surface debris may occur, these lead‐containing solids are easily removed from the toluene waste by standard filtration prior to disposal, ensuring environmentally responsible waste management.

Furthermore, while pseudo‐modules without electrodes were utilized in this study to facilitate high‐throughput material collection, this recovery framework is designed to be compatible with complete device architectures. In a full cell stack, the metal electrode would be detached via mechanical ‘lift‐off’ as toluene removes the underlying PCBM and BCP layers. This interfacial debonding causes physical detachment without chemical impact, ensuring the underlying perovskite remains intact.

### 2.3 Composition Analysis and Correction of Recovered Mixed‐Cation, Mixed‐Halide Perovskite

The recovered perovskite was expected to deviate in composition from the precursor salts due to cell processing. Therefore, we did not use it directly for device fabrication. Instead, we first quantified its composition in detail, in order to be able to restore it to a level comparable to the original precursor mixture. For comparison, devices fabricated from the uncorrected recovered perovskite are also discussed later alongside those made from the composition‐corrected recycled perovskite.

**FIGURE 5:**
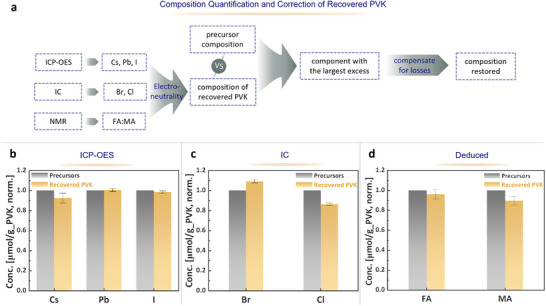
Composition analysis and correction of the recovered perovskite (“Recovered PVK”). All values are normalized to their respective values in the precursor mixture. (a) Workflow for composition determination and correction. (b) the concentrations of Cs, Pb, and I in the recovered PVK were measured by ICP‐OES. (c) Br and Cl measured by IC. (d) FA and MA content inferred from MAS NMR and the constraint of electroneutrality based on the measured cation and halide data.

As shown in the literature [21] and in the previous section, MAS NMR can reliably determine the FA:MA ratio. The remaining elements requiring quantification were Cs, Pb, I, Br, Cl, Ni, and K, with Ni and K being potential contaminants from the NiO_X_ layer. In principle, all these elements can be analyzed by inductively coupled plasma—optical emission spectroscopy (ICP‐OES); however, the halides require a specialized digestion matrix because they tend to form volatile species (e.g. HI/I_2_) in standard acidic media such as nitric acid [34, 35]. To obtain reliable results, an alkaline matrix is therefore necessary, and we found that ethylenediamine provided an effective solution for digesting these halides. Even so, ICP‐OES still offers a relatively poor detection limit for halogens. The fractional amounts of Br and Cl therefore require alternative characterization. Ion chromatography (IC) provides a suitable option due to its higher sensitivity than ICP‐OES for these halide species. Overall, ^1^H MAS NMR determined the FA:MA molar ratio (Figure S3), ICP‐OES quantified Cs, Pb, I, Ni, and K, and IC measured Br and Cl (Figure 5a). A complementary ^13^C MAS NMR is shown in Figure S4.

ICP‐OES results show the absence of K and the presence of trace Ni (11 ppm) in the recovered perovskite. The concentrations of the remaining components, normalized to the total sample mass, are summarized in Table 1, including the FA and MA contents deduced from the constraint of electroneutrality (explained in detail in the supporting information). These results reveal deviation in composition relative to the precursor mixture. To visualize these deviations, the component concentrations in the recovered perovskite were normalized to their respective values in the precursors (Figure 5b–d). Specifically, ICP‐OES revealed decreases in Cs and I and an increase in Pb (Figure 5b), while IC showed an increase in Br and a decrease in Cl (Figure 5c). The amounts of FA and MA both decreased, with MA exhibiting a more pronounced loss (Figure 5d). The apparent increases of Br and Pb reflect their lower depletion relative to other constituents rather than actual enrichment. Collectively, these analyses indicate that the recovered perovskite has an approximate composition of FA_0.839_MA_0.131_Cs_0.05_PbI_2.783_Br_0.15_Cl_0.086_.

**TABLE 1:** Comparison of the composition of the recovered perovskite and the precursors.

Component	Conc. in precursors (µmol g^−1^ total precursors)	Conc. in recovered PVK, (µmol g^−1^ PVK)	
		mean	standard error
I	4481.5	4408.7	55.9
Br	218.2	237.8	3.4
Cl	158.2	136.3	1.9
Cs	85.0	78.5	4.2
Pb	1578.9	1583.9	24.8
FA	1384.2	1329.5	64.8
MA	230.8	207.0	10.1

The deduced composition was subsequently re‐expressed in terms of the corresponding precursor compounds (Table 2), allowing a direct comparison between the recovered perovskite and the original precursor mixture to identify the component in the largest excess. Using that excess component as the reference, the theoretical amounts of the remaining components were calculated to satisfy the target composition—the composition of the precursors. The differences between the target and measured values yielded the compensation quantities required to restore the recovered perovskite to its original composition (Table 2).

**TABLE 2:** Composition of the recovered perovskite expressed in terms of the corresponding precursor compounds, and determination of the component compensation required to restore the original composition. The values are normalized to 1 g of recovered PVK.

Compound	MABr	MACl	CsI	FAI	PbBr_2_ ^a^	PbI_2_
Conc. in recovered PVK (µmol g^−1^)	70.7	136.3	78.5	1329.5	83.6	1500.4
Conc. in precursor mix (µmol g^−1^)	72.6	158.2	85	1384.2	72.8	1506.1
Ratio (recovered PVK/precursor, %)	97.3	86.1	92.3	96.0	114.8	99.6
Amount in recovered PVK (µmol)	70.7	136.3	78.5	1329.5	83.6	1500.4
Target amount for recycled PVK (µmol)	83.4	181.7	97.6	1589.5	83.6	1729.5
Compensation amount to reach target (µmol)	12.7	45.4	19.1	260.0	0.0	229.1

^a^ PbBr_2_ was used as the reference component for composition correction.

**FIGURE 6:**
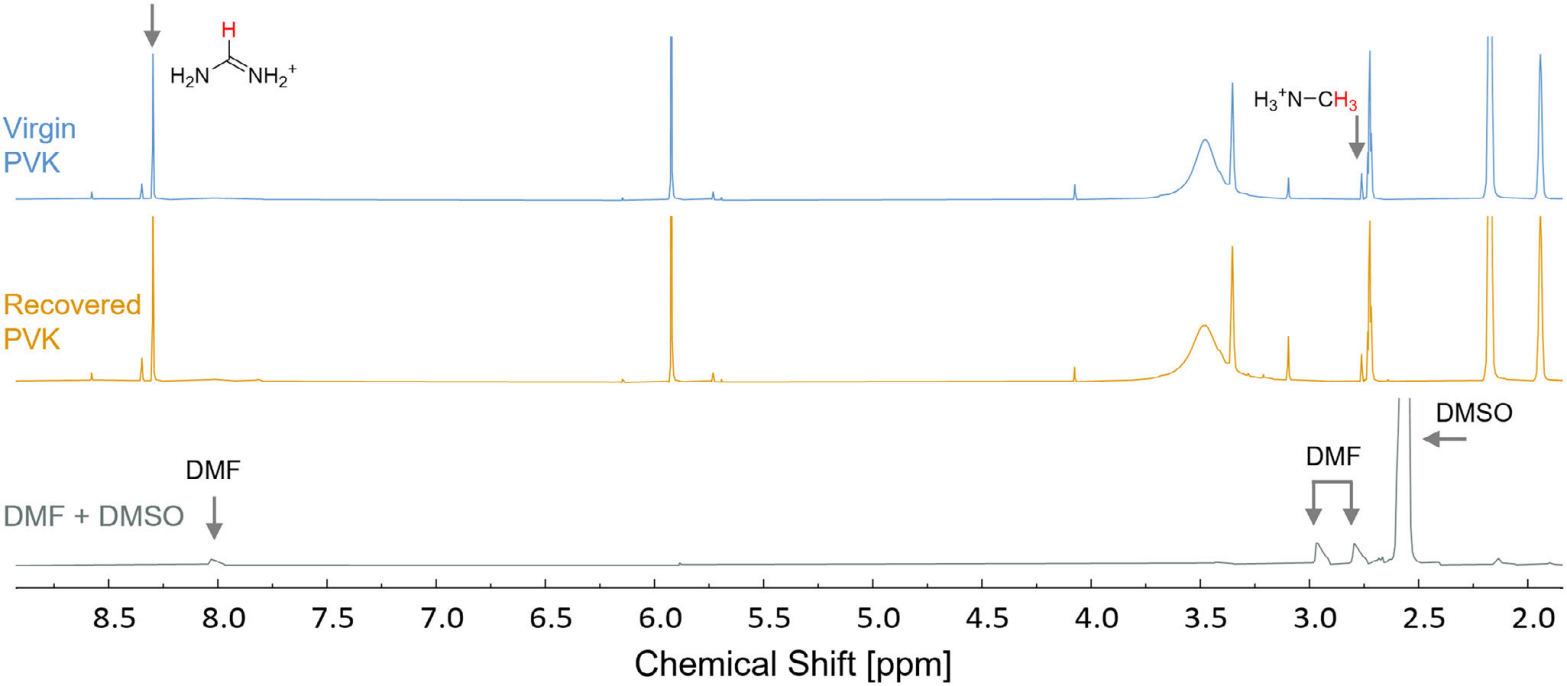
Solution ^1^H NMR comparison of Virgin PVK and Recovered PVK with reference spectrum of DMF and DMSO; all spectra were acquired in deuterated N‐methyl‐2‐pyrrolidone at 25°C, 500 MHz.

Before preparing the corrected recycled perovskite solution for cell fabrication, the possible presence of retained solvents (DMF and DMSO) should be considered, as these would influence the effective concentration of the solution. Therefore, a solution‐state NMR spectrum of the recovered perovskite was recorded and compared with those of the virgin perovskite, DMF, and DMSO, all using deuterated NMP as the solvent (Figure 6). The spectra confirm the absence of residual DMF and DMSO in the recovered perovskite. Furthermore, the recovered and the virgin perovskites exhibit identical spectral features, with no additional or unexpected signals observed in the recovered sample. The corresponding ^13^C solution‐state NMR spectra of virgin perovskite, recovered perovskite and deuterated NMP are provided in Figure S5, while the ^1^H NMR spectrum of deuterated NMP is shown in Figure S6.

### 2.4 Quality of Recycled Perovskite After Composition Correction

**FIGURE 7:**
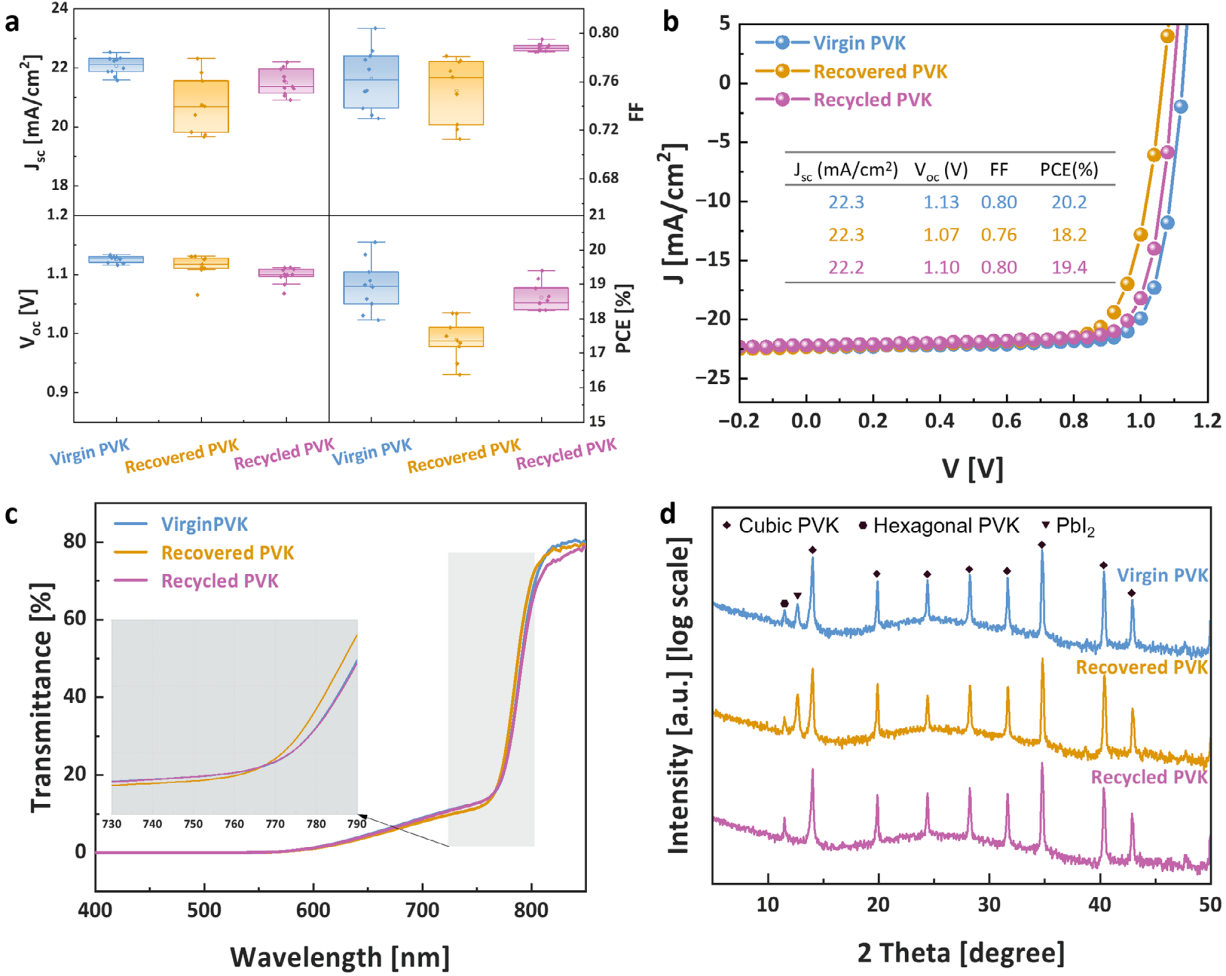
Device performance and characterization of composition‐corrected Recycled PVK. (a) Photovoltaic metrics (J_sc_, V_oc_, FF, and PCE) of solar cells made with Virgin PVK, Recovered PVK (direct product, without composition correction), and Recycled PVK (composition‐corrected product). (b) *J*–*V* characteristics of the best‐performing devices made with Virgin PVK, Recovered PVK, and Recycled PVK. (c) Transmittance spectra and (d) XRD patterns of Virgin PVK, Recovered PVK, and Recycled PVK.

The composition‐corrected recycled perovskite was subsequently used for solar cell fabrication. Figure 7a shows the corresponding device performance, compared with cells made from the virgin perovskite and uncorrected recovered perovskite. Without composition correction, the recovered perovskite yielded solar cells with lower efficiencies than those based on the virgin perovskite. Welch's *t* test (Table S2) confirms that the recovered PVK exhibited a significantly different PCE from the virgin PVK (*p* < 0.0001). The inferiority primarily originated from reduced current density. Profilometer measurements confirmed their film thickness was comparable (Figure S7), excluding thickness as the cause of the current loss. After composition correction, the cell performance, including current density, was largely restored. Welch's *t* test (Table S2) indicates the recycled perovskite exhibited efficiencies statistically indistinguishable from those of the virgin perovskite (*p* > 0.05). This result suggests that the applied composition correction enables the recycled hybrid perovskite to be reused in solar cell fabrication with performance matching that of the virgin material, thereby confirming the validity of the developed composition‐quantification approach. The *J*–*V* curves of the best‐performing devices (Figure 7b) also demonstrate an efficiency improvement after composition correction—from 18.2% for the recovered perovskite to 19.4% for the recycled perovskite—although the latter was marginally below the champion device based on the virgin material (20.2%). The minor performance deficiency originated from a reduced open‐circuit voltage. Because a trace Ni impurity (11 ppm) remained after composition restoration, Ni contamination was initially considered a possible contributor to the V_oc_ deficit. However, Welch's *t* test (Table S3) shows that the V_oc_ of devices based on recovered perovskite was statistically indistinguishable from that of virgin devices, whereas devices fabricated from recycled perovskite exhibited a lower V_oc_. Since both recovered and recycled perovskite contain comparable Ni levels, these results indicate that Ni contamination at this concentration is unlikely to be the dominant origin of the observed V_oc_ loss. We therefore consider alternative explanations. The slightly lower transmittance beyond 800 nm for the corrected sample might suggest additional sub‐gap absorption (Figure 7c), possibly arising from crystallization changes induced by the correction step. This could account for the small V_oc_ loss observed in the devices. Additionally, the transmittance spectra indicate a slightly shifted bandgap in the uncorrected recovered perovskite relative to the virgin one, which was restored after correction. XRD patterns (Figure 7d) show that the recovered perovskite exhibits peak positions identical to those of the virgin perovskite, indicating that both share the same crystal phases. However, the recovered perovskite shows a more pronounced PbI_2_ peak, consistent with the measured deficits in iodide and A‐site cations. After correction, the perovskite retained the same crystal phases as the virgin sample, and the PbI_2_ peak was largely suppressed or disappeared, implying that the correction was effective but may have slightly overcompensated for the initial deficiencies. Such minor overcorrection is reasonable, as the quantitative analysis combines results from several characterization techniques, each carrying inherent experimental uncertainty.

### 2.5 Impact of Full‐Composition Perovskite Recycling

We investigated the economic and environmental impacts of the developed full‐composition recycling approach for the hybrid perovskite. To assess the feasibility of scaling this process beyond laboratory experiments, we performed a material‐focused cost analysis based on an initial input of 1 kg of virgin perovskite precursors. Material costs were estimated using representative bulk laboratory pricing (e.g., 1 kg precursor batches and 25 L solvent containers), providing a consistent approximation of costs at an intermediate, pre‐industrial scale. On this basis, 1 kg of virgin perovskite precursor mixture costs 3029.1€ (Table S4). Starting from this input, the recovered material mass was estimated using the experimentally determined recovery yield of 89% together with an assumed 10% fabrication loss, included as a practical scale‐up allowance for processing inefficiencies rather than laboratory spin‐coating waste. The value of the recovered material was then calculated on a compound‐by‐compound basis using the recovered perovskite composition expressed in Table 2 as equivalent precursor compounds, giving a retained recovered value of 2420.5€ (Table S4). To maintain a constant 1 kg mass flow, fresh virgin precursors must be added to the recovered yield. This top‐up material serves two purposes: compensating for unavoidable physical losses during device fabrication and recovery, and restoring the composition. As detailed in Table S4, the amount required to correct composition drift is smaller than the amount needed to replace physical losses. Consequently, compositional correction is fully absorbed within the mandatory mass replacement and does not impose any additional material cost beyond the standard top‐up requirement. The resulting top‐up cost is therefore 608.6€.

To determine the net economic benefit of the recycling process, we further included the operating costs associated with recycling. As summarized in Table S5, these costs amount to 326.4€ for the batch recovered from the 1 kg virgin‐input basis and are dominated by solvent consumption (266.4€) and composition characterization (60.0€). The solvent cost reflects the process‐scale use of toluene, GBL, and ethyl acetate, with the required volumes estimated from working concentrations and material flow in each process step, as detailed in the supporting information. The characterization costs were calculated assuming thorough homogenization of the recovered material, such that a single representative analytical suite is sufficient to quantify the entire batch yielded from the 1 kg input. To reflect realistic scale‐up conditions, the cost model replaces the solid‐state MAS NMR used experimentally in this work with standard solution‐state NMR, a more accessible and cost‐effective alternative for determining FA:MA ratio [21].

**FIGURE 8:**
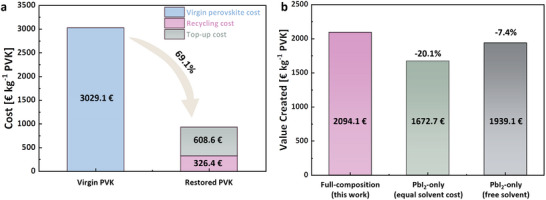
Economic benefits of full‐composition perovskite recycling. Analysis is normalized to an initial input of 1 kg of virgin precursors. (a) Cost reduction achieved by the full‐composition recycling approach (Restored PVK: recovered PVK and top‐up precursors to restore 1 kg PVK). (b) Net value created by full‐composition recycling compared to modeled PbI_2_‐only recycling, assuming either equivalent or zero solvent costs for the latter.

By adding these recycling costs (326.4€) to the required top‐up material expenses (608.6€), the effective cost of 1 kg of restored perovskite is 935.0€. Compared with the virgin‐material benchmark of 3029.1€ per kg, this corresponds to a net saving of 2094.1€ per kg, or a 69.1% cost reduction (Figure 8a; Table S6). While the exact magnitude of this saving may change upon further scale‐up, the direction of the economic effect is likely to remain favorable in industrial production. Importantly, the current recycling cost structure is dominated by solvent consumption, which is also one of the process components most amenable to reduction at scale through solvent recovery and reuse. At the same time, industrial implementation would introduce additional costs associated with process control, impurity management, and quality assurance, so the precise percentage saving will depend on the final process design. Nevertheless, because the economic benefit mainly arises from preserving the value of the recovered perovskite and thereby reducing the demand for virgin feedstock, rather than from laboratory‐specific processing conditions, the underlying advantage of full‐composition recycling is expected to persist.

We also compared the economic impact of full‐composition recycling with modeled PbI_2_‐only recycling scenarios (Table S7). Because PbI_2_‐only recycling does not require composition quantification, characterization costs were omitted in these models. For solvent expenses, we evaluated two cases: one assuming the same solvent cost as the full‐composition process and an idealized case assuming zero solvent cost. Under these conditions, full‐composition recycling generates a net value of 2094.1€, whereas PbI_2_‐only recycling generates 1672.7€ with equal solvent cost and 1939.1€ even when solvents are assumed to be free. This corresponds to a net value loss of 20.1% and 7.4%, respectively, relative to full‐composition recycling (Figure 8b). Notably, these scenarios still exclude the waste management cost associated with the unrecovered precursor elements, meaning their economic performance is likely overestimated.

Beyond the economic advantages, full‐composition recycling also offers environmental advantages over partial‐recovery routes, primarily through improving atom economy. While targeted lead sequestration addresses heavy‐metal toxicity, it leaves behind organic cations, cesium, and excess halides unrecovered. Without full‐composition recycling, these components are effectively lost to the waste stream and must be replaced by virgin feedstocks.

While the developed full‐composition recycling does not eliminate environmental burden—as it still requires chemical treatment and generates spent solvent streams and unrecovered fractions—the environmental trade‐off lies in the nature of the remaining burden. In the absence of full‐composition recycling, the residual waste contains dilute, high‐value components like iodine and cesium [13, 14, 15], whose subsequent reclamation from a complex waste mixture presents technical and energetic challenges. In contrast, full‐composition recycling shifts this burden toward process solvents. While these solvents require treatment, they are arguably more amenable to established industrial recovery techniques, such as distillation, than attempting to recover specific atomic components from a heterogeneous perovskite waste stream. Ultimately, by minimizing the continuous requirement for scarce primary feedstocks, this approach offers a viable pathway toward a more circular and resource‐efficient perovskite solar economy.

## 3 Conclusions

In summary, we developed a strategy for recycling mixed‐cation (FA, MA, Cs), mixed‐halide (I, Br, Cl) perovskites for solar cell fabrication. The compositional deviation of hybrid perovskite films from their precursors that occurred during solution processing inevitably leads to recovered materials whose compositions differ from those of their virgin counterparts. To address this, we first established a method to directly recover hybrid perovskites from precursor compounds without going through solution processing, which yielded solar cells with comparable efficiencies. Second, we developed a comprehensive approach to quantitatively determine the composition of hybrid perovskites by combining NMR, ICP‐OES, and IC. We subsequently extended the recovery approach from precursor mixtures to perovskite films within solar cell stacks and quantified the composition of the obtained recovered perovskite. Knowing this composition enabled us to restore the recovered perovskite to match that of the virgin precursors, thereby recovering cell performance to the level of the virgin material and validating the quantification approach. Our cost analysis at scale reveals that this full‐composition recycling reduces effective material costs by 69.1%. Furthermore, it generates at least 7.4% greater net value than PbI_2_‐only recycling while environmentally minimizing the loss of supply‐constrained elements (such as Cs and I) as secondary chemical waste. Beyond recycling, this diagnostic approach provides a powerful tool for reconstructing device degradation pathways, while the detection of migrated Ni impurities underscores the importance of designing perovskite architectures with recyclability in mind. Ultimately, this composition‐restored loop provides a sustainable blueprint for managing both heavy metals and scarce resources, complementing partial‐extraction strategies by targeting the full perovskite absorber.

## Author Contributions

Zhenni Wu contributed to conceptualization, data curation, formal analysis, validation, investigation, visualization, methodology, writing (original draft), and writing (review and editing). Katharina Dammer contributed to data curation, investigation, and writing (review and editing). Robert Skunde contributed to investigation and writing (review and editing). Mykhailo Sytnyk contributed to conceptualization, data curation, investigation, methodology, and writing (review and editing). Christian Göllner contributed to data curation, validation, investigation, and methodology. Fei Ding contributed to data curation, validation, formal analysis, methodology, and writing (review and editing). Juan S Rocha‐Ortiz contributed to data curation, validation, formal analysis, methodology, and writing (review and editing). Albert These contributed to investigation, formal analysis, and writing (review and editing). Balázs Imre contributed to investigation and methodology. Yanxue Wang contributed to investigation. Dorothea Wisser contributed to supervision, resources, and writing (review and editing). Christoph Brabec contributed to supervision, resources, funding acquisition, and project administration. Ian Marius Peters contributed to conceptualization, formal analysis, resources, supervision, funding acquisition, project administration, and writing (review and editing).

## Conflicts of Interest

The authors declare no conflicts of interest.

## Supporting information




**Supporting File**: adma73266‐sup‐0001‐SuppMat.docx.

## Data Availability

The data that supports the findings of this study are available in the supplementary material of this article.
