# Supplementary material for: Composition Restoration Enables Recycling of Mixed‐Cation, Mixed‐Halide Perovskites for Solar Cells
Source: Adv Mater. 2026 May 5;38(32):e73266. doi: 10.1002/adma.73266 (PMC13244800; doi:10.1002/adma.73266)
Supplement: Supplementary file 1 — Supporting File: adma73266‐sup‐0001‐SuppMat.docx. [file ADMA-38-e73266-s001.docx]

Supporting information

Composition Restoration Enables Recycling of Mixed-Cation, Mixed-Halide Perovskites for Solar Cells

Zhenni Wu^*1,2^, Katharina Dammer^1^, Robert Skunde^1,2^, Mykhailo Sytnyk^2^, Christian Göllner^2^, Fei Ding^3^, Juan S Rocha-Ortiz^2^, Albert These^1,2^, Balázs Imre^1,2^, Yanxue Wang^1,2^, Dorothea Wisser^3^, Christoph Brabec^1,2^, and Ian Marius Peters^*1,2^

^1^Friedrich-Alexander-Universität Erlangen-Nürnberg, Department of Material Science, Materials for Electronics and Energy Technology (i-MEET), 91058 Erlangen, Germany

^2^Forschungszentrum Jülich, Helmholtz Institute Erlangen-Nürnberg for Renewable Energies (HI ERN), 91058 Erlangen, Germany

^3^Friedrich-Alexander-Universität Erlangen-Nürnberg, Erlangen Center for Interface Research and Catalysis (ECRC), 91058 Erlangen, Germany

*Corresponding authors. Email: [z.wu@fz-juelich.de](mailto:z.wu@fz-juelich.de); [i.peters@fz-juelich.de](mailto:i.peters@fz-juelich.de)

# Experimental Procedures

## Materials

Indium tin oxide (ITO)/glass substrates were purchased from Liaoning Yike Precision New Energy Technology Co., Ltd. Hellmanex III was purchased from Hellma Analytics. Acetone (≥ 99%, technical) and isopropanol (≥ 98%, technical) for cleaning was purchased from VMR. Deionized water was obtained with an in-house filter.

NiO nanoparticle solution were purchased from Lumtec. Nickel(II) nitrate hexahydrate (99.999%), Ethylenediamine (synthesis grade, ≥99%), Ethylene glycol (ReagentPlus, ≥99%) were all purchased from Sigma Aldrich. KCl (99%) were purchased from Thermo Fisher.

[4-(3,6-Dimethyl-9H - carbazol-9-yl)butyl]phosphonic acid (Me-4PACz, >99%) and lead iodide (PbI_2_, 99.99%) were purchased from TCI Co., Ltd. Methylammonium bromide (MABr, >99.99%), methylammonium chloride (MACl, >99.99%), formamidinium iodide (FAI, >99.99%) were purchased from Greatcell Solar Materials. Cesium iodide (CsI, 99.999%) and lead bromide (PbBr_2_, 99.999%) were purchased from Sigma Aldrich.

[6,6]-Phenyl-C61-butyric acid methyl ester (PCBM) were purchased from Nano-C. Bathocuproine (BCP) were purchased from Xi’an Yuri Co., Ltd. N,N-dimethylformamide (DMF, extra dry, 99.8%), dimethyl sulfoxide (DMSO, extra dry, 99.7%), chlorobenzene (extra dry, 99.8%), isopropanol (extra dry, 99.5%) and toluene (anhydrous, 99.8%) were acquired from Thermo Fisher. Ethyl acetate (anhydrous, 99.8%) were purchased from VMR. γ-Butyrolactone (GBL, 99%) and N-Methyl-d3-2-pyrrolidinone-d6 (98 atom% D) was purchased from Sigma Aldrich. GBL was purged with argon before use to remove oxygen. Lead ICP standard, cesium ICP standard, periodic table mix 1 for ICP (including nickel and potassium), bromide standard solution, chloride standard solution, iodide standard solution and nitric acid (65%) were all purchased from Sigma Aldrich. These standard solutions were used for inductively coupled plasma-optical emission spectroscopy (ICP-OES) and ion chromatography (IC). Tetramethylsilane (TMS, ≥99.9%) was purchased from Carl Roth for solution-state NMR.

## Solution preparation

The NiO_x_ sol–gel solution was prepared by dissolving nickel(II) nitrate hexahydrate (5 g) with ethylene glycol (17.195 mL). Ethylenediamine (1.145 mL) was then added dropwise under stirring, and the mixture was stirred overnight at room temperature. An aliquot (10 mL) of the resulting solution was mixed with 185 μL of KCl (100mg mL^-1^ in H_2_O) to afford a 1 M NiO_x_ stock solution. This stock was diluted with ethylene glycol and ethylenediamine (same ratio as above) to prepare a 0.1 M NiO_X_ sol–gel solution. This recipe was adapted from literature. ^1^

The Me-4PACz solution (0.5 mg mL^-1^) was prepared in DMF by vortexing for 2 minutes.

Following literature,^2^ the FA/MA/Cs perovskite solution was prepared by dissolving MABr (6.7 mg), MACl (8.8 mg), CsI (18.2 mg), PbBr_2_ (22 mg), FAI (196.1 mg) and PbI_2_ (572 mg) in DMF:DMSO (4:1 v/v; total solvent 998 μL to reach 1.3 M or 865 μL for 1.5 M) and stirred at 60 ^o^C for 1 hour. This solution was filtered with a 0.2 μm PTFE filter before use.

PCBM solutions in chlorobenzene (20 or 25 mg mL^-1^) and BCP solutions in isopropanol (0.5 mg mL^-1^) were stirred at 60 ^o^C overnight to ensure complete dissolution. Before use, all solutions were filtered through 0.2 μm PTFE filters.

## Device fabrication

The solar cells made with virgin perovskite and those with recycled perovskite use the same fabrication parameters as follows. The functional solar cells could be fabricated with spin coating with a commercial automated setup called ‘Spinbot’ from SCIPRIOS, or manually but with different parameters. Both methods are presented below.

25 x 25 mm^2^ ITO/glass substrates were sequentially sonicated in 2% Hellmanex III, DI H_2_O, acetone and isopropanol for 15 min each, dried under a N_2_ stream and treated with UV-ozone for 40 min.

Parameters for automated deposition:

A NiO­_x_ solution (Lumtec) was statically dispensed onto the substrate, spun at 2000 rpm for 30 s, and annealed at 150 ^o^C for 30 min. The following layers before electrode were deposited in a N_2_-filled glovebox. Me-4PACz solution was statically coated at 3000 rpm for 25 s and annealed at 100 ^o^C for 10 min. Next, the FA/MA/Cs perovskite solution (1.3 M) was dispensed and spun at 400 rpm for 15 s and 4000 rpm for 17 s, then 170 μL of chlorobenzene was dynamically cast, followed by continued spinning at 4000 rpm for the 15s. The samples were then annealed at 120 ^o^C for 20 min. PCBM solution was dynamically coated by spinning at 1000 rpm followed by solution dispensing and then continuing at the same speed for 60 s, and annealed at 80 ^o^C for 10 min. Subsequently, BCP was dynamically coated at 5000 rpm for 30 s and annealed at 80 ^o^C for 5 min.

Parameters for manual deposition:

NiO_x_ sol-gel solution was statically dispensed onto the ITO substrate and spun at 4000 rpm for 20 s with an acceleration rate of 1000 rpm/s, followed by annealing at 100 ^o^C for 5-30 min and subsequently at 300 ^o^C for 1h under ambient conditions. Then the substrates were transferred into a N_2_-filled glovebox. Me-4PACz was coated onto the substrates statically at 3000 rpm for 25 s and a ramping time of 2s and then annealed at 100 ^o^C for 10 min. The FA/MA/Cs perovskite solution (1.5 M) was further statically coated at 1000 rpm for 5s, then 4000 rpm for 30s (ramping time from 1000 rpm to 4000 rpm is 2s). 300 μL of Chlorobenzene was dispensed onto the substrate when there was 15s remaining. The samples were then annealed at 100 ^o^C for 50 min. PCBM solution was dynamically coated at 1000 rpm for 60s and then annealed at 80 ^o^C for 10 min. BCP solution was dynamically coated at 5000 rpm for 30 sand then annealed at 80 ^o^C for 5 min.

Lastly, an approximately 100 nm thick Ag layer was evaporated onto the masked samples, resulting in an effective cell area of 0.063 cm^2^.

## Pseudo-module fabrication

Glass substrates (50 x 50 mm^2^) were treated with UV-ozone for 10 min. The pseudo-modules were all doctor-bladed. 30 μL of 0.1 M NiO_x_ sol-gel solution was bladed at 5 mm s^-1^ using a 100 μm blade gap on a 45 ^o^C coating bed, followed by annealing at 100 ^o^C for 5–30 min and subsequently at 300 ^o^C for 1h under ambient conditions. All subsequent layers were all deposited inside a N_2_-filled glovebox. Me-4PACz solution (30 μL) was coated at 5 mm s^-1^ with a 100 μm gap at 30 ^o^C, quenched under N_2_ at 1 bar for 1 min, and annealed at 100 ^o^C for 10 min. FA/MA/Cs perovskite solution (80 μL, 2M) was coated at 30 mm s^-1^ with a 250 μm gap at 40 ^o^C. The wet films were immediately quenched under N_2_ at 1.8 bar until they turned fully dark, immersed in ethyl acetate for 1 min, and annealed at 100 ^o^C for 50 min. PCBM solution (40 μL) was coated at 10 mm s^-1^ with a 100 μm gap on a 40 ^o^C bed, quenched under N_2_ at 1 bar for 1 min, and annealed at 80 ^o^C for 10 min. Finally, BCP solution (40 μL) was coated under the same conditions as PCBM, but annealed at 80 ^o^C for 5 min.

## Recovery

The recovery process was performed in a N_2_-filled glovebox. The modules were firstly immersed in toluene for 1 min, and the process was repeated twice to completely remove PCBM and BCP layers. Subsequently, the perovskite layer was dissolved using oxygen-free GBL. 1 mL fresh GBL in total was applied for 10 modules via a gravity-assisted rinse approach with solvent reuse to minimize solvent consumption and keep concentration high for subsequent crystallization. Specifically, each module was placed inclined in a beaker. GBL was pipetted onto the surface, allowing it to flow across the perovskite layer. The perovskite layer dissolved almost immediately and then drained by gravity, accumulating in the beaker. For the first module, we applied 300 μL of fresh GBL to generate an initial perovskite/GBL solution. A final rinse with 70 μL of fresh GBL was then applied to recover residual solution from the substrate. For the subsequent modules, the dissolution step was performed using the previously accumulated perovskite/GBL solution, and only the final 70 μL rinse was fresh for each module. The resulting solution was filtered through a 0.2 μm PTFE filter, and ethyl acetate—4 times the volume of the filtrate—was added. The mixture was vortexed for 1 min and centrifuged at 3500 rpm for 5 min to collect the precipitated perovskite. The supernatant was discarded, and the precipitate was washed twice with ethyl acetate to remove residual GBL. Lastly, the recovered perovskite was dried at 70 ^o^C under vacuum.

## Characterization

The current-voltage characteristics of the solar cells were recorded using a Keithley 7001 Switch system, which automatically selected different cells on the sample, and a Keysight B2901A Precision Source/Measure Unit to apply controlled voltages and record the resulting currents). Measurements were performed under 100 mW cm^-2^ AM 1.5G illumination from a Wavelabs Sinus-70 light engine calibrated using a crystalline Si reference cell. The voltage was scanned from 1.2 to -0.2 V in the reverse direction.

The transmittance spectra were measured on a PerkinElmer Lambda 950 spectrophotometer equipped with a 150 mm integrating sphere. Perovskite films were spin-coated on bare glass under the same conditions as for solar cell fabrication.

X-ray diffraction (XRD) patterns were collected in a standard ex situ Bragg-Brentano configuration using a PANalytical X’pert powder diffractometer equipped with filtered Cu-K*α* radiation (λ = 1.54178 Å) and an X’Celerator solid-state stripe detector operated at 40 kV and 30 mA. The diffraction patterns were recorded over a 2θ range of 10–50^o^, with a step size of 0.0394^o^. All perovskite films were prepared on bare glass substrates under the same conditions as used for solar cell fabrication.

^1^H Magic Angle Spinning (MAS) NMR spectra were recorded on a 11.74 T (500 MHz ^1^H Larmor frequency) Agilent DD2 spectrometer. The samples were spun at a MAS rate of 12 kHz in a zirconia rotor. A DEPTH sequence was used to suppress probe background signals, consisting of a π/2 pulse of 2.5 μs followed by two π pulses of 5.0 μs, and phase cycled according to a combined “EXORCYCLE” and “CYCLOPS” scheme^3^ 16 scans were accumulated with a recycle delay of 110 s (corresponding to 5 x T_1_, determined previously from saturation recovery experiments) to ensure complete relaxation. The perovskite samples were prepared by spin coating under the same conditions as used for solar cell fabrication, followed by mechanical scraping to collect the material. Approximately 10-20 mg of material from 8-15 perovskite films were required for one NMR sample. Deconvolution of ^1^H DEPTH MAS NMR spectra was done using DMFit.^4^

^13^C direct excitation spectra were recorded at a MAS rate of 12 kHz for all samples. A π/2 pulse of 2.5 μs, followed by 100 kHz SPINAL-64 decoupling on the ^1^H channel during the relaxation delay and detection was used. Recycle delays were set to 100 s,^5^ and 600-800 scans were recorded.

The resonance at 0 ppm of sodium 2,2-dimethyl-2-sila-pentane-5-sulfonate (DSS) was used as chemical shift reference.

The concentrations of Pb, Cs, I, Ni, and K in the recycled perovskite were quantified via ICP-OES, PerkinElmer Optima 8300 DV. For Ni, K, Pb, Cs, 12.5 mg of perovskite was digested in 5 mL of 2% HNO_3_. For iodide, 5 mg of the recycled perovskite was digested in 5 mL of 5% ethylenediamine. Three replicate samples were analyzed for both types. Additionally, reference standards (PbI_2_, CsI, and FAI for Pb, Cs and I, respectively) were measured to determine correction factors for the quantitative analysis. Calibration solutions were prepared from purchased standard stock solutions. For Ni and K, concentrations of 0.2, 0.05, and 0.01 mg L^-1^ were used. For Cs, concentrations of 50, 20 and 2 mg L^-1^ were used, and for Pb, 800, 500 and 50 mg L^-1^. For I^-^, concentrations of 640, 400 and 40 mg L^-1^ were used.

The concentrations of Br and Cl in the recycled perovskite were quantified via IC, Dionex ICS-5000+DC from Thermo Scientific. 6 mg of perovskite was digested in 5 mL of 5% ethylenediamine. Three replicate samples were measured. Reference standards (MACl and PbBr_2_ for Cl and Br, respectively) were measured to determine correction factors. For calibration solutions, concentrations of 100, 50, 25, and 5 mg L^-1^ were used.

Solution-state NMR spectroscopy was performed on a JEOL ECA-500 spectrometer, with a magnetic field strength of (¹H: 500 MHz; ¹³C: 125 MHz). Chemical shifts were reported in parts per million (ppm) and referenced to residual protic impurities in the solvent for ¹H NMR, and to the deuterated solvent signal for ¹³C NMR. For NMP-d_9_, the spectra were referenced to tetramethylsilane (TMS) at 0.00 ppm.

The film thickness was measured using a KLA P-7 profilometer. The perovskite films were prepared on bare glass following the same procedure as used for cell fabrication. The step edges for profilometry were created by scraping the film with a blade.

## Deduction of FA and MA using electroneutrality

The concentrations of Cs^+^, Pb^2+^, I^-^, Br^-^ and Cl^-^ in the recovered perovskite were determined by ICP-OES and IC. The impurity Ni, at only 11 ppm, was negligible. Assuming electroneutrality among FA^+^, MA^+^, Cs^+^, Pb^2+^, and the halides, and using the FA:MA molar ratio obtained from MAS NMR, the concentrations (μmol g^-1^) of FA and MA were calculated according to:

| $n \left( FA \right)=x \left( FA \right)\times\left( n\left( I \right)+n\left( Br \right)+n\left( Cl \right)-n\left( Cs \right)-2n(Pb \right))$ | (1) |
| --- | --- |
| $n \left( MA \right)=x \left( MA \right)\times\left( n\left( I \right)+n\left( Br \right)+n\left( Cl \right)-n\left( Cs \right)-2n(Pb \right))$ | (2) |

where $n$ denotes the amount of substance per gram of sample and $x$ is the molar fraction of FA or MA within FA and MA.

## Statistical Analysis

A Welch’s t test^6^ was applied to determine whether the means of two groups differ significantly, assuming unequal variances. The test statistic, which measures how far apart the two sample means are in units of the standard error of the difference, is calculated as:

| $t= \frac{\bar{X_{1}}+\bar{X_{2}}}{\sqrt{\frac{s_{1}^{2}}{n_{1}}+\frac{s_{2}^{2}}{n_{2}}}}$ | (3) |
| --- | --- |

where $\bar{X}$ is the sample mean, $s$ the standard deviation, and $n$ the sample size.

The degrees of freedom (df), which determines the shape of the t-distribution, are estimated using the Welch-Satterthwaite equation:

| $df= \frac{{(\frac{s_{1}^{2}}{n_{1}}+\frac{s_{2}^{2}}{n_{2}})}^{2}}{\frac{{({s_{1}^{2}}/{n_{1}})}^{2}}{n_{1}-1}+\frac{{({s_{2}^{2}}/{n_{2}})}^{2}}{n_{2}-1}}$ | (4) |
| --- | --- |

The calculated $|t|$ value is compared with the critical value from the t-distribution corresponding to the chosen significance level (typically 0.05) and the calculated $df$.

If $|t|$ exceeds the critical value, the difference between the two means is considered statistically significant.

Alternatively, a p-value can be computed, representing the probability of observing the obtained t-statistic (or a more extreme value) under the null hypothesis that the two group means are equal.

A p-value can be obtained using statistical software such as Excel or OriginLab. If the p-value is less than 0.05, the null hypothesis is rejected, indicating a statistically significant difference; otherwise, the difference is not statistically significant.


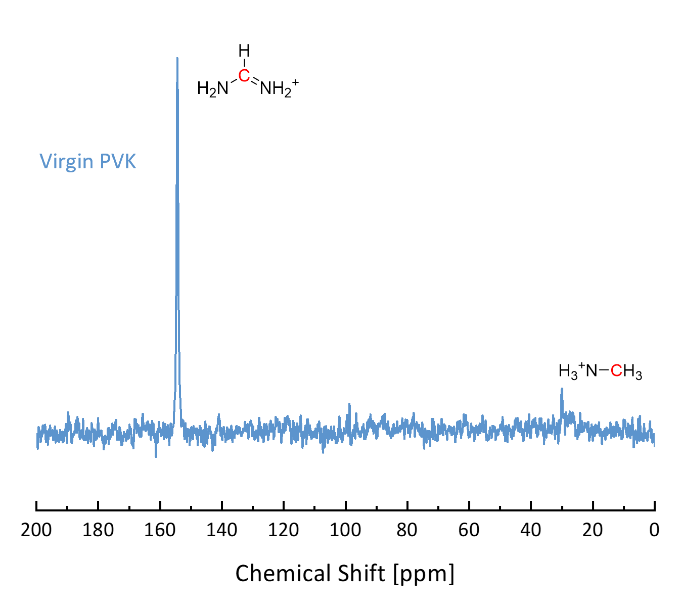


Figure S1. ^13^C MAS NMR spectrum of virgin PVK.


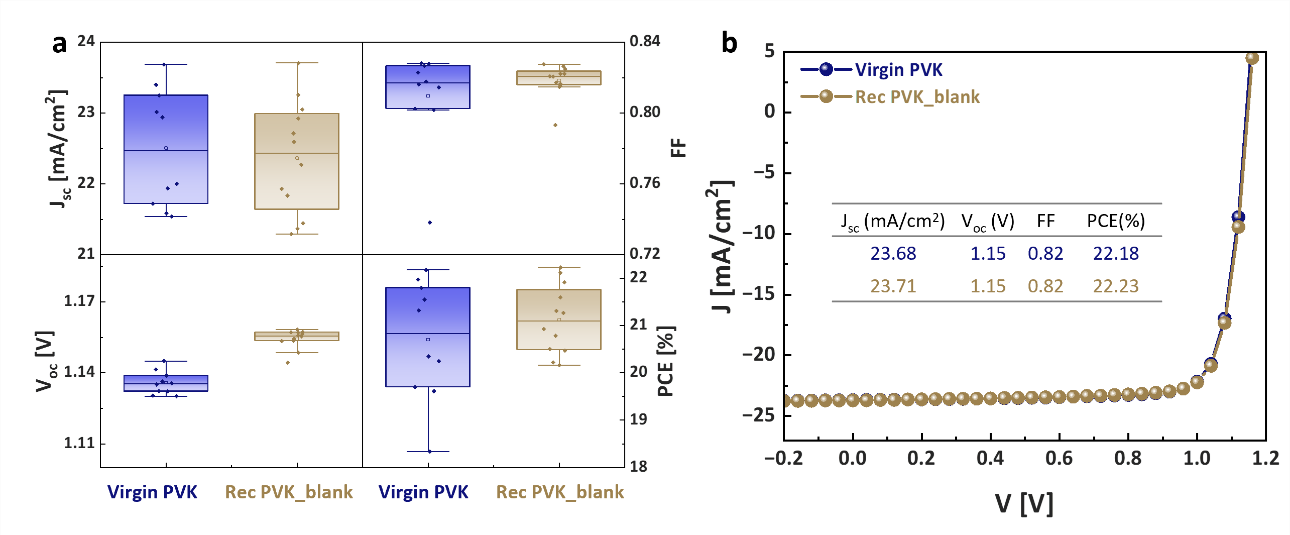


Figure S2. Effect of PEAI passivation on V_oc_ in Rec PVK_blank vs Virgin PVK. A Welch’s $t$ test indicates no statistically significant difference between the two groups ($p$ > 0.05). The device architecture used was ITO/NiO_x_/Me-4PACz/PVK/PEAI/PCBM/BCP/Ag.


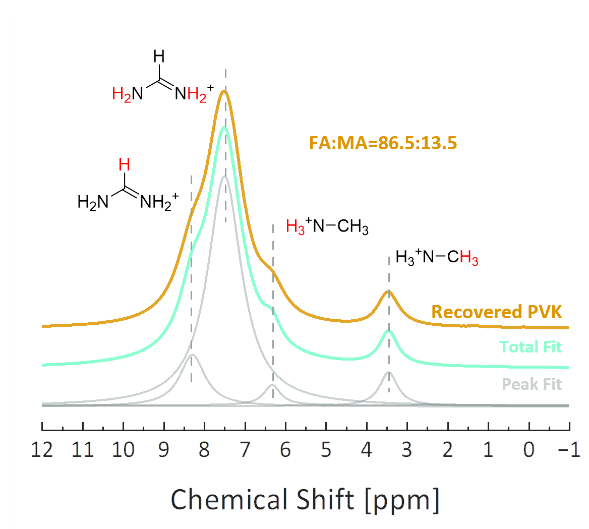


Figure S3. ^1^H MAS NMR of Recovered PVK with deconvolution.


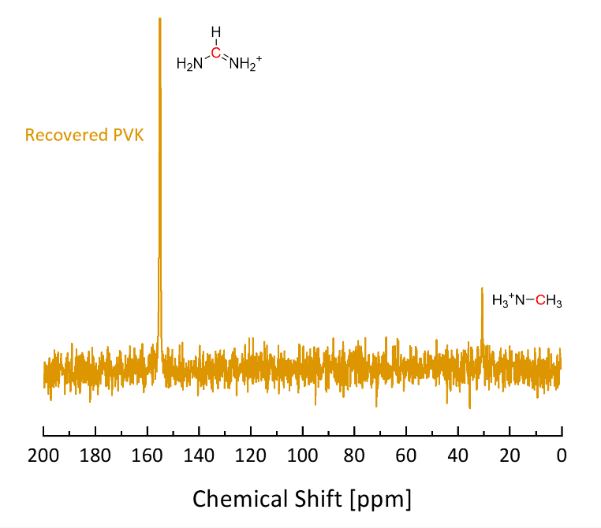


Figure S4. ^13^C MAS NMR spectrum of the recovered PVK.


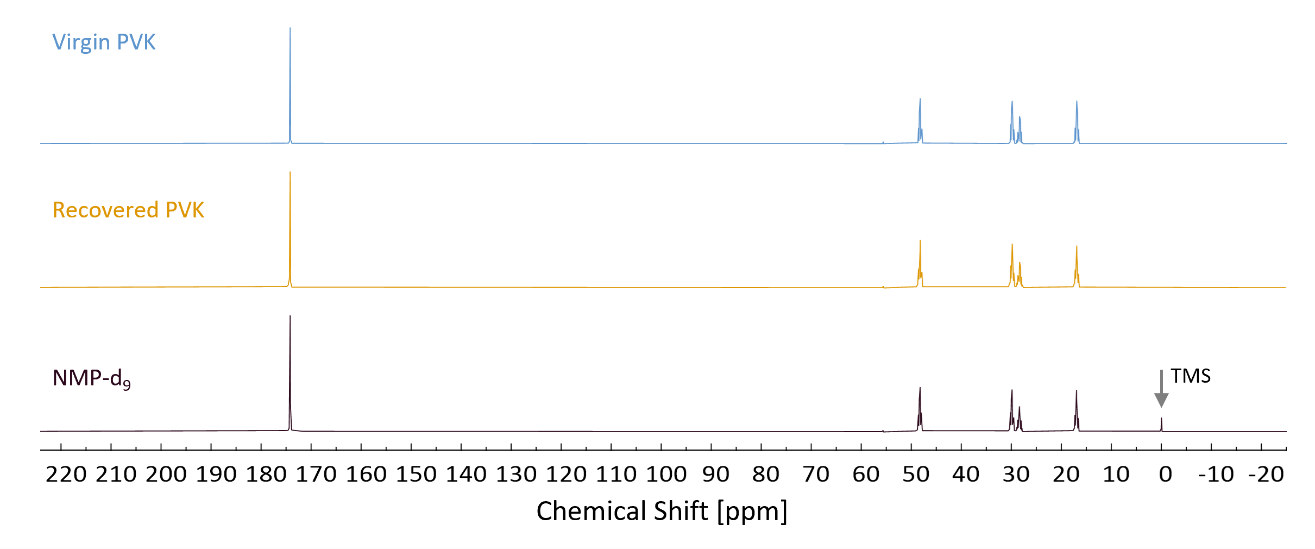


Figure S5. ^13^C solution-state NMR spectra of virgin PVK, recovered PVK and deuterated N-methyl-2-pyrrolidone, 25 ^o^C, 125 MHz.


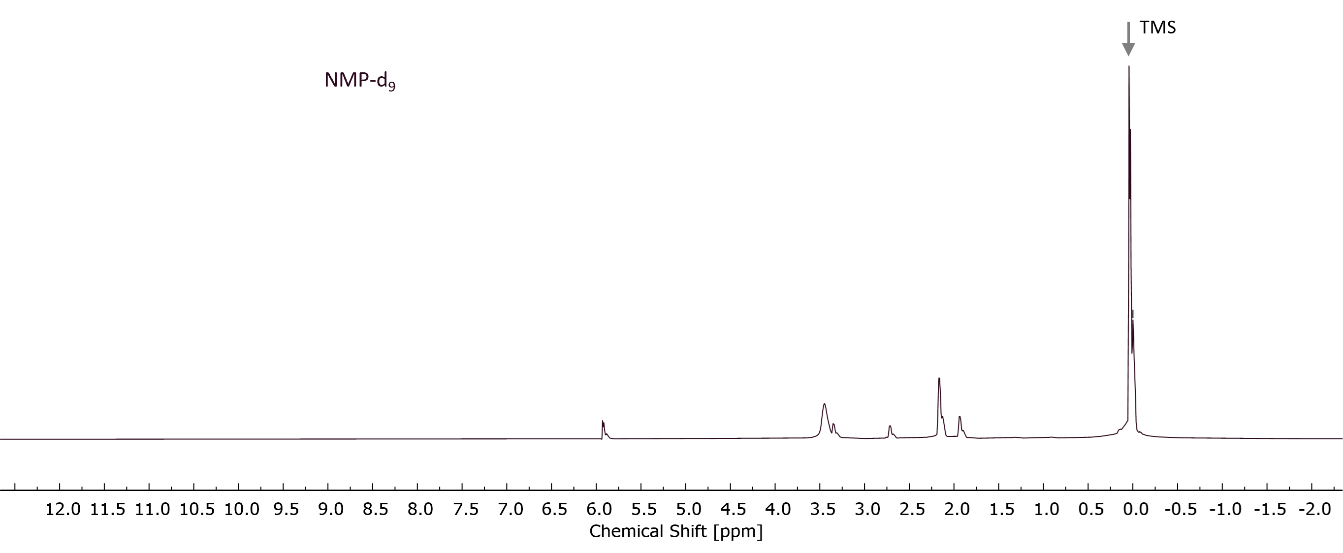


Figure S6. Solution ^1^H NMR of deuterated N-methyl-2-pyrrolidone at 25 ^o^C, 500 MHz.


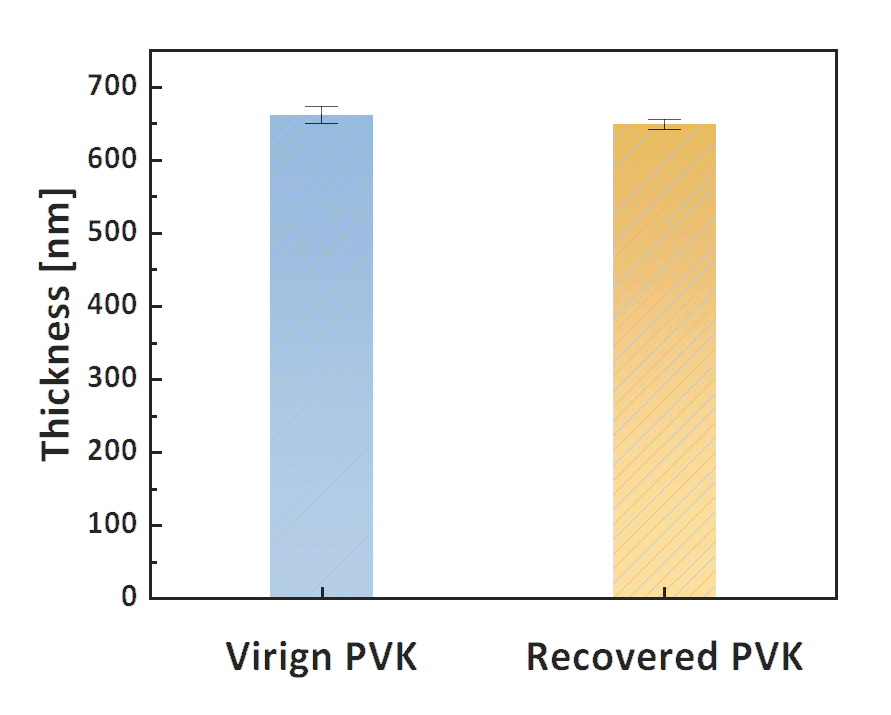


Figure S7. Film thicknesses of the virgin and recovered perovskite films.

Table S1. Welch’s t test results for the power conversion efficiencies (PCE) of Virgin PVK and Rec PVK_Blank

| Test statistic (t) | Degrees of Freedom (df) | Critical value (α=0.05) | P-value |
| --- | --- | --- | --- |
| 1.88 | 21 | 2.08 | 0.074 |

Table S2. Results of Welch’s t tests comparing the PCEs of virgin PVK with recovered and recycled PVK.

|  | Test statistic (t) | Degrees of Freedom (df) | Critical value (α=0.05) | P-value |
| --- | --- | --- | --- | --- |
| Virgin PVK vs Recovered PVK | 5.13 | 17 | 2.11 | < 0.0001 |
| Virgin PVK vs Recycled PVK | 1.31 | 13 | 2.16 | 0.2120 |

Table S3. Results of Welch’s t tests comparing the V_oc_s of virgin PVK with recovered and recycled PVK.

|  | Test statistic (t) | Degrees of Freedom (df) | Critical value (α=0.05) | P-value |
| --- | --- | --- | --- | --- |
| Virgin PVK vs Recovered PVK | 1.7 | 9.18 | 2.26 | 0.1209 |
| Virgin PVK vs Recycled PVK | 6.4 | 14.07 | 2.15 | < 0.0001 |

Table S4. Precursor material costs, recovered material value, and top-up cost required to restore 1 kg of perovskite, normalized to 1 kg of virgin perovskite.

| Com-pound | Price^a^ | Virgin mass | Virgin cost | Recover-ed content | Recover-ed mass^b^ | Recover-ed value | mass to restore comp-osition | Top-up mass | Top-up cost |
| --- | --- | --- | --- | --- | --- | --- | --- | --- | --- |
|  | € kg^-1^ | g | € | mol g^-1^ | g | € | g | g | € |
| MABr | 680 | 8 | 5.5 | 70.7 | 6.3 | 4.3 | 1.1 | 1.8 | 1.2 |
| MACl | 379 | 11 | 4.1 | 136.3 | 7.4 | 2.8 | 2.5 | 3.3 | 1.3 |
| CsI | 2790 | 22 | 61.6 | 78.5 | 16.3 | 45.6 | 4.0 | 5.8 | 16.1 |
| FAI | 1582 | 238 | 376.6 | 1329.5 | 183.1 | 289.7 | 35.8 | 54.9 | 86.9 |
| PbBr_2_ | 5657 | 27 | 151.1 | 83.6 | 24.6 | 139.0 | 0.0 | 2.1 | 12.1 |
| PbI_2_ | 3500 | 694 | 2430.2 | 1500.4 | 554.0 | 1939.1 | 84.6 | 140.3 | 491.1 |
| Total |  |  | 3029.1 |  |  | 2420.5 |  |  | 608.6 |

^a^: material prices were based on 1 kg bulk quantities sourced from Greatcell Solar (MABr, MACl, FAI), Sigma Aldrich (CsI, PbBr_2_), and TCI (PbI_2_). Because 1 kg commercial pricing was unavailable for PbBr_2_, its price was extrapolated using a 10% learning rate per volume doubling according to previous literature.^7^

^b^: To estimate the total recovered mass from 1 kg of virgin perovskite, our calculations incorporate both the recovery yield (89%) and material losses during device fabrication. At industrial scales, high-waste methods like spin coating will likely be replaced by scalable techniques such as slot-die coating, which can achieve material waste below 1%.^8^ However, to account for practical inefficiencies, such as imperfect yields, fluid filtration, rinsing, and system priming, we assumed a 10% material loss during fabrication.

Table S5. Recycling costs for 1 kg virgin perovskite.

| Consumables/Process | Usage^c^ | | Cost rate^d^ | | Total cost (€) |
| --- | --- | --- | --- | --- | --- |
| *Solvents* |  | |  | |  |
| Toluene | 3.6 L | | 18.9 € / L | | 67.0 |
| GBL | 1.4 L | | 36.9 € / L | | 49.8 |
| Ethyl acetate | 8.1 L | | 18.4 € / L | | 149.5 |
| Solvents Subtotal |  | |  | | 266.4 |
| *Composition quantification* | |  | |  | |
| NMR | 1 batch | | 10.0 € | | 10.0 |
| ICP-OES (3 elements) | 1 batch | | 30.0 € | | 30.0 |
| IC (2 elements) | 1 batch | | 20.0 € | | 20.0 |
| Characterization subtotal |  | |  | | 60.0 |
| Total process cost |  | |  | | 326.4 |

^c^: Solvent consumption volumes for toluene and GBL were calculated using a 70% of their maximum working concentrations: the theoretical saturation limit of PCBM in toluene (16.3 mg/mL),^9^ and an experimentally validated concentration (952 mg/mL) of perovskite in GBL derived from our recovery trials.

Because toluene is used to extract the PCBM layer, its required volume depends on the mass of PCBM ($m\left( PCBM \right)$). This was determined geometrically by scaling the mass of the perovskite layer ( $m\left( PVK \right)$) using their respective material densities ($\rho$) and film thicknesses ($t$):

| $m\left( PCBM \right)=m\left( PVK \right)\times\frac{\rho(PCBM)\times t(PCBM)}{\rho\left( PVK \right)\times t(PVK)}$ | (5) |
| --- | --- |

Normalized to 1 kg of virgin perovskite precursors with an assumed 10% production loss, the perovskite film mass is $m\left( PVK \right)=0.9 kg$. Using material densities of $\rho\left( PCBM \right)=1.5 g {cm}^{-3}$ ^10^ and $\rho\left( PVK \right)=4.1 g {cm}^{-3}$ (based on FAPbI_3_)^11^, alongside layer thickness of $t\left( PCBM \right)=80 nm$ ^12^ and $t\left( PVK \right)=650 nm$ (Figure S7), the extracted mass of PCBM is approximately 40.5 g, which directly dictates the required toluene volume.

The total consumption of ethyl acetate is estimated as the sum of the volume required for anti-solvent crystallization (four times the GBL volume) and two subsequent purification washes, with each wash cycle assumed to use a volume equivalent to the initial GBL.

^d^: Solvent prices were based on 25 L bulk quantities sourced from Sigma Aldrich (GBL) and Carl Roth (toluene and ethyl acetate). Because 25 L commercial pricing was unavailable for GBL, its price was extrapolated using a 10% learning rate according to previous literature.^7^

Here, NMR refers to ^1^H solution NMR, which can determine FA:MA ratio at substantially lower cost than MAS NMR. Analytical costs were estimated using a uniform unit fee of 10 € per measurement output (one ^1^H solution NMR, each ICP element, and each IC ion/analyte), selected as a rounded value consistent with the order of magnitude of published German academic service tariffs despite differing billing schemes.^13, 14^

Table S6. Cost reduction induced by recycling, normalized to 1 kg of virgin perovskite.

| Metric | Amount (€) |
| --- | --- |
| Cost of 1 kg Virgin Perovskite | 3029.1 |
| Recycling costs | 326.4 |
| Top-up cost to restore 1 kg perovskite | 608.6 |
| Effective cost of 1 kg restored perovskite (recycled + top-up) | 935.0 |
| Net Savings per kg | 2094.1 |
| Cost Reduction (%) | 69.1% |

Table S7. Economic impact of full composition vs PbI_2_-only recycling scenarios, normalized to 1 kg of virgin perovskite.

| Metric | Scenario 1: Full Composition Recycling | Scenario 2: PbI₂-Only (Same Solvent Cost) | Scenario 3: PbI₂-Only ("Free" Solvents) |
| --- | --- | --- | --- |
| Value of Recovered PbI₂ | 1,939.1 € | 1,939.1 € | 1,939.1 € |
| Value of Other Materials (FAI, PbBr₂, CsI, MABr, MACl) | 481.4 € | 0.0 € | 0.0 € |
| [A] Gross Recovered Value | 2420.5 € | 1939.1 € | 1939.1 € |
| Solvent Costs | 266.4 € | 266.4 € | 0.0 € |
| Characterization Costs | 60.0 € | 0.0 € | 0.0 € |
| [B] Total Recycling Costs | 326.4 € | 266.4 € | 0.0 € |
| [A - B] Net Value Created | 2094.1 € | 1672.7 € | 1939.1 € |
| Absolute Value Lost (compared to Scenario 1) | - | -421.4 € | -155.0 € |
| Net Value Loss (%) | 0% | -20.1% | -7.4% |

References

(1) Niu, B.; Liu, H.; Huang, Y.; Gu, E.; Yan, M.; Shen, Z.; Yan, K.; Yan, B.; Yao, J.; Fang, Y.*; et al.* Multifunctional hybrid interfacial layers for high-performance inverted perovskite solar cells. *Adv. Mater.* **2023**, *35*, e2212258.

(2) Tan, Q.; Li, Z.; Luo, G.; Zhang, X.; Che, B.; Chen, G.; Gao, H.; He, D.; Ma, G.; Wang, J.*; et al.* Inverted perovskite solar cells using dimethylacridine-based dopants. *Nature* **2023**, *620*, 545–551.

(3) Bendall, M. R.; Gordon, R. E. Depth and refocusing pulses designed for multipulse NMR with surface coils. *J. Magn. Reson.* **1983**, *53*, 365–385.

(4) Massiot, D.; Fayon, F.; Capron, M.; King, I.; Le Calvé, S.; Alonso, B.; Durand, J.-O.; Bujoli, B.; Gan, Z.; Hoatson, G. Modelling one- and two-dimensional solid-state NMR spectra. *Magn. Reson. Chem.* **2002**, *40*, 70–76.

(5) Franssen, W. M. J.; Bruijnaers, B. J.; Portengen, V. H. L.; Kentgens, A. P. M. Dimethylammonium incorporation in lead acetate based MAPbI3 perovskite solar cells. *ChemPhysChem* **2018**, *19*, 3107–3115.

(6) WELCH, B. L. The generalisation of student’s problems when several different population variances are involved. *Biometrika* **1947**, *34*, 28–35.

(7) Chang, N. L.; Ho-Baillie, A. W. Y.; Vak, D.; Gao, M.; Green, M. A.; Egan, R. J. Manufacturing cost and market potential analysis of demonstrated roll-to-roll perovskite photovoltaic cell processes. Sol. Energy Mater. Sol. Cells **2018**, 174, 314–324.

(8) Chaturvedi, N.; Gasparini, N.; Corzo, D.; Bertrandie, J.; Wehbe, N.; Troughton, J.; Baran, D. All slot-die coated non-fullerene organic solar cells with PCE 11%. Adv. Funct. Mater. **2021**, 31, 2009996.

(9) Wang, W.; Guo, S.; Herzig, E. M.; Sarkar, K.; Schindler, M.; Magerl, D.; Philipp, M.; Perlich, J.; Müller-Buschbaum, P. Investigation of morphological degradation of P3HT:PCBM bulk heterojunction films exposed to long-term host solvent vapor. J. Mater. Chem. A **2016**, 4, 3743–3753.

(10) Liu, H.-J.; Jeng, U.-S.; Yamada, N. L.; Su, A.-C.; Wu, W.-R.; Su, C.-J.; Lin, S.-J.; Wei, K.-H.; Chiu, M.-Y. Surface and interface porosity of polymer/fullerene-derivative thin films revealed by contrast variation of neutron and X-ray reflectivity. *Soft Matter* **2011**, *7*, 9276–9282.

(11) Stoumpos, C. C.; Malliakas, C. D.; Kanatzidis, M. G. Semiconducting tin and lead iodide perovskites with organic cations: phase transitions, high mobilities, and near-infrared photoluminescent properties. *Inorg. Chem.* **2013**, *52*, 9019–9038.

(12) Wang, J.; Zardetto, V.; Datta, K.; Zhang, D.; Wienk, M. M.; Janssen, R. A. J. 16.8% Monolithic all-perovskite triple-junction solar cells via a universal two-step solution process. *Nat. Commun.* **2020**, *11*, 5254.

(13) Leibniz Universität Hannover, Institut für Anorganische Chemie. *Anlage Preise Kleine Moleküle und Metabolomik*; Stand 01.04.2025; Hannover, Germany, 2025. <https://www.naturwissenschaften.uni-hannover.de/fileadmin/naturwissenschaften/NAT_Forschung/CoreNAT/Kleine-Molekuele_Metabolomik/Anlage_Preise_Kleine_Molekuele_und_Metabolomik_Stand_01.04.2025.pdf> (accessed March 10, 2026).

(14) Ruhr-Universität Bochum, Geowissenschaften. *Nutzungsordnung Hydrochemisches Labor*; Bochum, Germany, <https://www.geo.ruhr-uni-bochum.de/sites/default/files/media/file/NutzungsOrdnung%20Hydrochemisches%20Labor_0.pdf> (accessed March 10, 2026).
